# Identification and validation of expression and functions of ferroptosis-related gene HILPDA in early-onset preeclampsia placentas

**DOI:** 10.3389/fimmu.2025.1627057

**Published:** 2025-08-11

**Authors:** Qianghua Wang, Xuegu Wang, Jiaojiao Fei, Chuanyue Jiang, Yafen Tao, Nana Yang, Huijuan Chen, Chengli Dou, Biao Ding, Danli Du, Xiang Li

**Affiliations:** ^1^ Laboratory of Department of Infectious Diseases, First Affiliated Hospital of Bengbu Medical University, Bengbu, Anhui, China; ^2^ Reproductive Medicine Center, First Affiliated Hospital of Bengbu Medical University, Bengbu, Anhui, China; ^3^ Molecular Diagnosis Center, Anhui Province Key Laboratory of Clinical and Preclinical Research in Respiratory Disease, First Affiliated Hospital of Bengbu Medical University, Bengbu, Anhui, China

**Keywords:** early-onset preeclampsia, HILPDA, trophoblast migration, immune infiltration, ferroptosis

## Abstract

**Background:**

Early-onset preeclampsia (EOPE) is a severe form of preeclampsia that mainly contributes to maternal and perinatal morbidity and mortality worldwide. This study aimed to systematically analyze the expression and function of ferroptosis-related gene HILPDA in EOPE placentas.

**Methods:**

We included five transcriptomic datasets (GSE148241, GSE44711, GSE74341, GSE114691, GSE10588) downloaded from the Gene Expression Omnibus (GEO) in this study. Using differential expression analysis, weighted gene co-expression network analysis (WGCNA), and machine learning models (LASSO, SVM-RFE, Random Forest), We identified hub genes and diagnostic biomarkers. We performed functional enrichment (GO and KEGG) and immune infiltration analysis to elucidate molecular mechanisms. Experimental validation included Western blot on clinical placental samples and siRNA-mediated knockdown in HTR-8/SVneo trophoblasts to assess migration.

**Results:**

We observed HILPDA upregulation and confirmed its diagnostic accuracy (AUC=0.71) in EOPE placentas. Functional analysis revealed HILPDA-associated enrichment in immune regulation (leukocyte migration, MHC complexes) and cellular processes (collagen organization, HIF-1 signaling). Through WGCNA, we identified 171 HILPDA-associated DEGs. Machine learning prioritized PART1 as diagnostic biomarkers. Immune profiling highlighted HILPDA’s correlation with activated dendritic cells, neutrophils and resting mast cells. Experimentally, we confirmed HILPDA up-regulation in EOPE placentas and its critical role in suppressing trophoblast migration.

**Conclusions:**

Our study establishes HILPDA as a central mechanistic regulator involved in placental immune dysregulation and trophoblast migration in EOPE pathogenesis. The identified biomarkers PART1 and HILPDA-associated pathways may offer novel diagnostic and therapeutic targets for EOPE management, which contribute to reduce maternal morbidity and prevent perinatal mortality in this catastrophic pregnancy syndrome.

## 1 Introduction

Preeclampsia (PE), a common obstetric complication specific to human, accounts for approximately 70,000 maternal and 500,000 fetal deaths annually, with a global incidence ranging from 5% to 7% of all pregnancies (1). PE is characterized by new-onset hypertension and proteinuria in the maternal host after the 20th week of gestation, often accompanied by organ damage such as hepatic and renal impairment. Based on the timing of clinical symptoms onset, PE is classified into two categories (2): early-onset preeclampsia (EOPE, <34 gestational weeks) and late-onset preeclampsia (LOPE, ≥34 gestational weeks). Compared to LOPE, EOPE exhibits an expeditious onset, swift progression, and an unfavorable prognosis. Despite its prevalence, the pathogenic mechanisms of PE remain unclear, and effective treatments and preventive strategies, aside from timely pregnancy termination, are lacking (3).

Recent studies have established a strong correlation between the occurrence of EOPE and placental ferroptosis. Ferroptosis is a novel form of programmed cell death, triggered by the catalysis of cell membrane unsaturated fatty acid lipids by ferrous iron or lipoxygenase. Unlike apoptosis, necrosis, and autophagy, ferroptosis is iron-dependent and closely associated with oxidative stress (4). During pregnancy, the increased maternal iron demand and reduced response to environmental iron exposure make pregnant individuals prone to iron overload, resulting in pregnancy complications (5). Previous studies have shown that serum iron and ferritin levels are significantly higher in patients with EOPE compared to those with LOPE and control groups, indicating a strong positive correlation with the severity of PE (5). This is supported by other studies that showed the significant role of ferroptosis in the pathogenesis of EOPE (6, 7).

In preeclamptic patients, abnormal remodeling of uterine spiral arteries leads to placental ischemia and hypoxia, causing oxidative stress and elevated reactive oxygen species (ROS) in the placenta. Growing evidence suggests that iron-induced ferroptosis, driven by iron overload and placental hypoxia, plays a significant role in the pathogenesis of PE. In animal models of PE, inhibiting ferroptosis alleviates PE symptoms and improves fetal survival rates (8). Decreasing ferritin levels early in pregnancy or increasing them later exacerbates PE symptoms by promoting ferroptosis (9). A recent study indicates that ferroptosis caused by ferritin light chain deficiency can induce PE by disrupting the remodeling of uterine spiral arteries (7). While ferroptosis provides a mechanistic framework for placental damage, the upstream triggers remain incompletely defined.

Hypoxia-inducible lipid droplet-associated protein (HILPDA) is a small protein induced by hypoxia, consisting of 63 amino acids (10). It plays a critical regulatory role in the storage of triglycerides and the supply of intracellular fatty acids, thus promoting lipid storage in liver cells, macrophages, and cancer cells (10). A recent study suggests that the hypoxia-induced HIF-2α subtype can reshape the cell membrane’s polyunsaturated lipids via HILPDA, thereby increasing the sensitivity of clear cell carcinoma cells to ferroptosis (11). However, the role of HILPDA in PE is still unknown.

In this study, we identified the ferroptosis-related gene HILPDA as a potential biomarker for EOPE. Using integrated computational and bioinformatics strategies, we analyzed HILPDA-related genes and signaling pathways in PE placentas. Additionally, we validated the expression of HILPDA in EOPE placentas and examined its regulatory role in cell migration within trophoblast cells *in vitro*. Our findings underscore the significant involvement of HILPDA in EOPE and offer new insights and potential therapeutic targets for managing this severe obstetric complication.

## 2 Methods

### 2.1 Data acquisition

In this study six public transcriptomic datasets GSE148241 (12), GSE44711 (13), GSE74341 (14), GSE114691 (15), GSE10588 (16) and GSE75010 (17) (**Supplementary Table S1**) were downloaded from the Gene Expression Omnibus database (GEO) (18) (https://www.ncbi.nlm.nih.gov/geo/) based on: 1) Sample size ≥ 5 per group (EOPE vs. control); 2) Clinically confirmed EOPE diagnosis per ACOG criteria; 3) Exclusive use of placental tissue (no cell lines or other tissues). Platform-specific normalization pipelines were implemented: RNA-seq datasets (GSE114691) underwent median-of-ratios normalization using DESeq2, while microarray datasets (GSE148241, GSE10588, GSE44711, GSE74341, GSE75010) were processed with limma quantile normalization.

The ferroptosis-related gene sets were acquired from FerrDb (http://www.zhounan.org/ferrdb/index.html) (19). FerrDb contained 258 experimentally validated FRGs at time of access (2022). Database expansions since then include indirect regulators not analyzed here. The full list of FRGs was shown in **Supplementary Material**.

### 2.2 EOPE placenta collection

Placental specimens were obtained from six normal normotensive controls and six EOPE cases (determined through power analysis and clinical constraints) after obtaining informed consent (**Supplementary Table S2**). EOPE diagnosis followed the American College of Obstetricians and Gynecologists (ACOG) criteria, requiring new-onset hypertension (systolic BP ≥140 mmHg/diastolic ≥90 mmHg) with proteinuria (≥300 mg/24h) or end-organ dysfunction, occurring after 20 weeks and before 34 weeks of gestation. Exclusion criteria include: pregnant women diagnosed with cardiovascular disease, metabolic syndrome, endocrine disorders, liver, or kidney diseases, as well as cases involving fetal malformations or chromosomal abnormalities. The samples were then cleansed with balanced salt solution to eliminate any blood, flash-frozen in liquid N_2_ within 20 min of delivery, and stored at -80°C for ≤6 months before analysis for western blot analyses. The Ethics Committee of the First Affiliated Hospital of Bengbu Medical College granted approval for this study under protocol number ([2024]KY023).

### 2.3 Cell culture and treatment

HTR-8/SVneo cells were cultured in RPMI-1640 complete medium (RPMI-1640 medium containing 10% fetal bovine serum (FBS, 164210, Procell Life Science & Technology, Wuhan, Hubei, China), 100 U/ml penicillin, and 100 μg/ml streptomycin) at 37 °C with 5% CO2. Cells were seeded in the RPMI-1640 complete medium for 24 hours and then transfected with siRNAs or negative control siRNA using Lipo3000 (Thermo Fisher Scientific, USA) for 48 hours and the knockdown efficiency was confirmed by real-time quantitative PCR and western blot. The siRNA sequences are shown as **Supplementary Table S3**.

### 2.4 Real-time quantitative PCR

Total RNA from cells was extracted using TRIzol (Invitrogen, Carlsbad, CA). The concentration and purity of the extracted RNA were evaluated with a NanoDrop™ One/OneC (Thermo Fisher Scientific). cDNA was generated using a Novo-Scrip^®^ Plus All-in-one 1st Strand cDNA Synthesis Kit (E042-01B, Novoprotein, China) at 42°C for 5 minutes, 50°C for 15 minutes, and 75°C for 5 minutes. The qRT-PCR was performed using NovoStart^®^ SYBR qPCR SuperMix Plus (E167-01A, Novoprotein, China) and the LightCycler 480 (Roche, Basel, Switzerland) was used for amplifications and quantifications. Primers were validated through melt curve analysis (single-peak confirmation) and the sequences were shown as follows (5′–3′):

HILPDA-F: AAGCATGTGTTGAACCTCTAC
HILPDA-R: TGTGTTGGCTAGTTGGCTTCT
GAPDH-F: CAATATGATTCCACCCATGGCA
GAPDH-R: GCATCGCCCCACTTGATTTT

### 2.5 Western blot

Cultured cells or pre-homogenized tissues were lysed with ​ice-cold RIPA buffer (Beyotime, P1005, China) containing 1× protease inhibitor cocktail​(Beyotime, P0013B, China) with gentle agitation at 4°C for 30 min, followed by​centrifugation at 12,000×g for 15 min at 4°C to collect clarified lysates. The total protein was quantified using the Total Protein Assay Kit (Beyotime, P0012, China) and subsequently denatured. Proteins (50 μg per lane) were separated on SDS–polyacrylamide gels and then transferred onto polyvinylidene fluoride (PVDF) membranes. The membranes were incubated overnight at 4°C with the following primary antibodies: anti-HILPDA (sc-376704, 1:100, Santa Cruz) and anti-β-actin (LF201, 1:5000, Epizyme, Shanghai, China). After being washed with TBST, the membranes were incubated for 2 hours at room temperature with HRP-labeled Goat Anti-Mouse IgG secondary antibodies (LF101, 1:5000, Epizyme, Shanghai, China). Protein bands were detected using a ChemiDoc Imaging System (UVP ChemStudio, Analytik Jena, Germany) and quantified with ImageJ software.

### 2.6 Wound healing assay

HTR-8/SVneo cells transfected with siHILPDA or controls (scrambled siRNA) were scratched using 200-μl pipette tips when cells were cultured to approximately 90% confluency. After gently washing the cells twice with phosphate-buffered saline (PBS), the wells were replenished with serum-free RPMI 1640 medium (Thermo Fisher Scientific, USA) to eliminate proliferation bias. Microscopic images of the wound areas were captured at 0 h (baseline) and 24 h post-wounding using an inverted phase-contrast microscope (Motic, Xiamen, China). Quantitative analysis of cell migration was performed by measuring the residual scratch area at both time points with ImageJ software. The migration rate was calculated using the following formula: [(Initial area - Final area)/Initial area] × 100%.

### 2.7 Differentially expressed genes analysis and the screening and evaluation of candidate genes

The limma and DESeq2 packages in R were used to identify differentially expressed genes (DEGs). For RNA-seq count data, genes were filtered by requiring ≥10 raw counts in a minimum of k samples, with k defined as the sample size of the smallest experimental group (EOPE or CON). DEGs were defined by p-value <0.05 and |log_2_FC| > 1. In GSE114691 datasets, all patients were stratified into HILPDA-high and HILPDA-low groups based on median HILPDA expression, with DEGs identified using p-value <0.05 and |FC| > 1. The VennDiagram package in R was used to identify common genes among DEGs in the GSE148241, DEGs in GSE44711 and ferroptosis-related gene sets. Receiver operating characteristic (ROC) curves and the area under the ROC curve (AUC) were calculated to evaluate the diagnostic potential of the candidate genes.

### 2.8 Machine learning algorithms for identification of candidate diagnostic biomarkers

Three machine learning models (LASSO, SVM-RFE, Random Forest) were trained on z-score normalized expression of the 171 WGCNA-selected genes. 10-fold cross-validation with 5 repeats was used for hyperparameter tuning and performance evaluation. The least absolute shrinkage and selection operator (LASSO) regression, random forest and support vector machine-recursive feature elimination (SVM-RFE) were performed on GSE114691(n=61: 21 EOPE/40 controls). Model discrimination was quantified by mean cross-validated AUC. External validation was performed on GSE75010.

The LASSO regression, implemented via the R package *glmnet* (*v4.1-8)*, employs L1 regularization to achieve high-dimensional data compression and feature selection. In this analysis, the algorithm effectively distilled 171 candidate genomic features into 5 non-zero biomarkers through an optimized λ parameter (lambda.1se = 0.186) determined via 10-fold cross-validation.

The random forest algorithm was implemented using the *randomForest* (*v4.7-1.2)* package in R. Initial model training employed 1,000 decision trees (ntree = 1000) to capture feature interactions, with subsequent optimization using out-of-bag (OOB) error analysis. The optimal mtry (number of features per split) was determined via iterative testing (1–20 features), selecting the value minimizing OOB error (mtry = 14).

The SVM-RFE algorithm integrates L2-norm regularization with backward feature elimination to identify optimal feature subsets for predictive modeling. Implemented via the *e1071 (v1.7-16)* and *caret (v7.0-1)* R packages. A 10-fold cross-validation (nfold = 10) was applied to partition samples into training/validation subsets.

### 2.9 Weighted gene co-expression network analysis

To identify the co-expression gene module associated with EOPE and the screened candidate gene HILPDA, WGCNA was performed in GSE114691 datasets using the WGCNA package in R. Specifically, hierarchical clustering analysis was first performed using *hclust* function, and then the suitable soft threshold power was determined using the pickSoftThreshold function and an adjacency cutoff value of 0.9. We selected a soft-thresholding power of 19 based on rigorous evaluation of the scale-free topology criterion. This power represented the minimum value achieving satisfactory scale-free model fit (R² = 0.92) while maintaining biological interpretability. Finally, co-expression gene modules were constructed by set the minimum module size of 30.

### 2.10 Functional enrichment analysis

To perform functional enrichment analysis of screened genes associated with EOPE and HILPDA expression, Functional enrichment was performed using the clusterProfiler package (v4.10.0). Gene ontology (GO) terms (20) and Kyoto Encyclopedia of Genes and Genomes (KEGG) (21) analyses were conducted with enrichGO and enrichKEGG package respectively. The GO analysis encompassed biological processes (BPs), cellular components (CCs), molecular functions (MFs), and pathway analyses. The Benjamini-Hochberg method was employed to adjust the P values, with adjusted *P*-values < 0.05 set as the threshold for significance.

### 2.11 Immune infiltration analysis

Immune cell infiltration analysis was performed using ​CIBERSORT (v1.03) (22).​​ The LM22 gene signature​ (downloaded from CIBERSORT portal) containing 547 genes defining 22 human immune cell types. Gene expression matrices were formatted with unique gene symbols​ in row names (duplicates resolved by ​mean expression). We ensured ​no missing values​ by excluding genes with missing data and enabled ​quantile normalization (QN=TRUE)​​ to minimize batch effects across samples. To investigate immunomodulatory relationships, Spearman’s rank correlation analysis was systematically performed between HILPDA expression and immune subsets.

### 2.12 Gene set enrichment analysis

In the GSE114691 datasets, gene set enrichment analysis (GSEA) (23) was performed between low- and high-HILPDA expression (dichotomized at median expression) to identify the biological process (GO term) and pathways (KEGG) associated with HILPDA expression. Gene set permutations to compute log2 fold change. GSEA analysis was performed using the gseGO and gseKEGG methods of the clusterprofiler package in R.

### 2.13 Statistical analysis

Continuous variables between two groups were compared using unpaired Student’s t-tests​, implemented in GraphPad Prism 9. Bioinformatics analyses were conducted using R software (v4.3.1). The chi-squared tests were used for the analysis of count data between two groups. All data are presented as mean ± standard deviation (SD). A *P*-value of less than 0.05 was considered statistically significant.

## 3 Results

### 3.1 Identification of HILPDA as a differentially expressed ferroptosis-related gene and diagnostic biomarker in EOPE placentas

**Figure 1:**
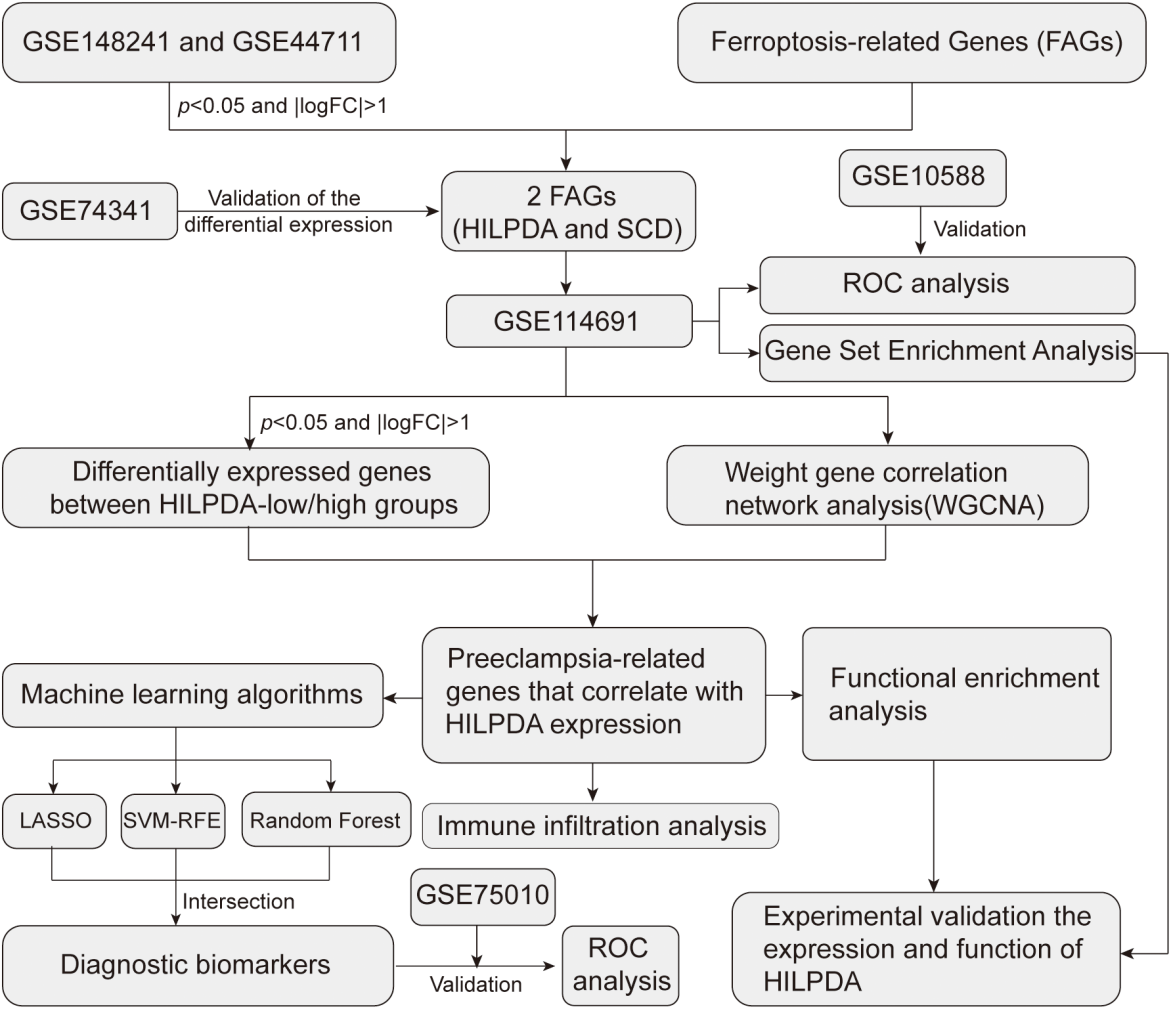
The workflow of the present study.

**Figure 2:**
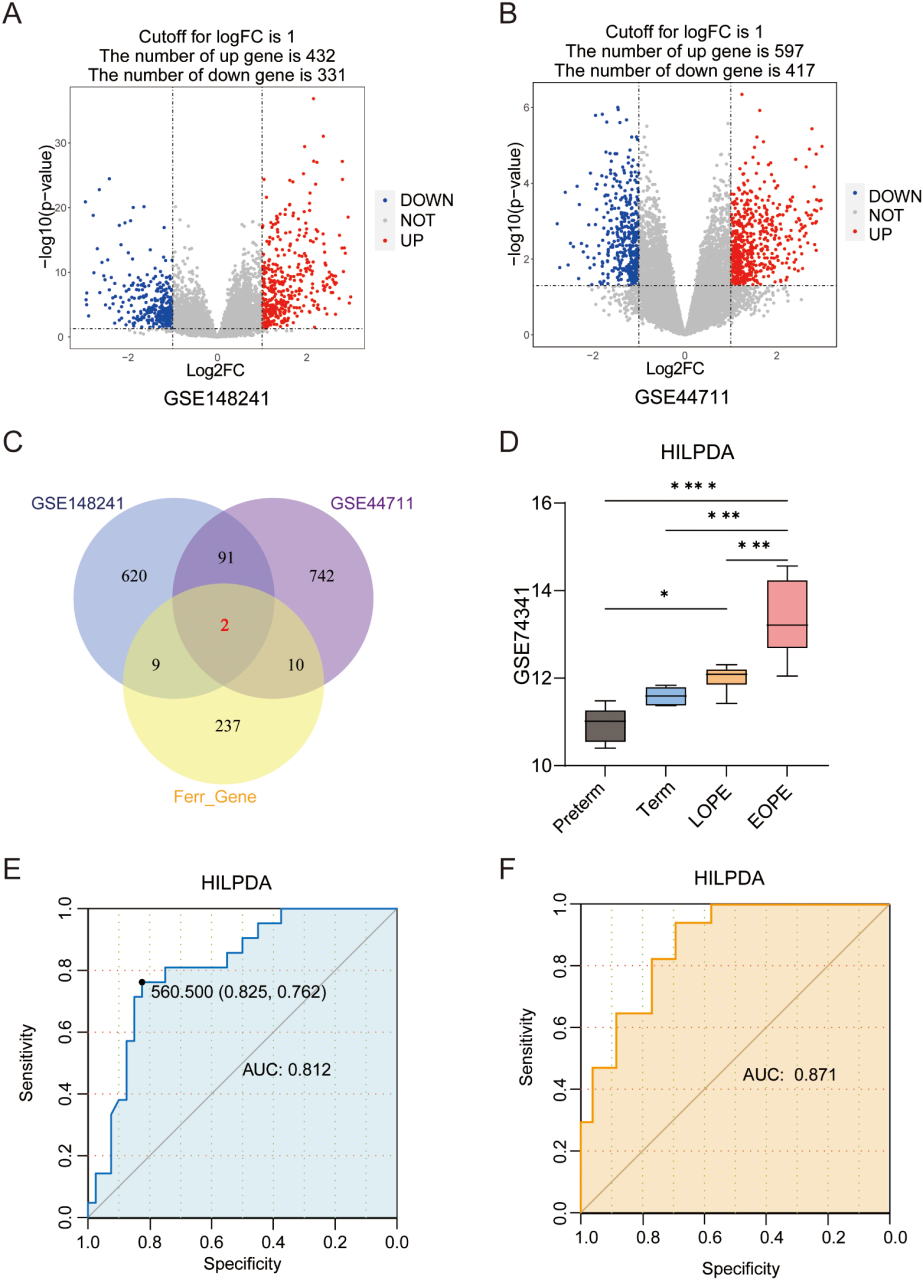
Differentially expressed ferroptosis-related genes (FRGs) and the prognostic value of HILPDA in EOPE. **(A)** The volcano plot of differentially expressed genes (DEGs) in the placental samples of EOPE patients from GSE148241 and GSE44711(n); **(B)** The volcano plot of differentially expressed genes (DEGs) in the placental samples of EOPE patients from; **(C)** Intersection of DEGs from GSE148241 and GSE44711 with FRGs. **(D)** The expression of HILPDA in placentas from preterm, term, LOPE and EOPE patients in GSE74341. The ROC curve of HILPDA for diagnosing EOPE in GSE114691 **(E)** and GSE10588 **(F)** dataset. ^*^ *P* less than 0.05; ^***^ *P* less than 0.001; ^****^ *P* less than 0.0001.

To screen for the differentially expressed FRGs in EOPE, differential expression analysis between the placenta samples from EOPE and control patients was performed in GSE148241(32 controls, 9 EOPE) and GSE44711 (8 controls, 8 EOPE) datasets. The study workflow was shown in **Figure 1**. Volcano plots identified 763 DEGs (432 upregulated) in GSE148241 placentas (**Figure 2A**) and 1,014 DEGs (597 upregulated) in GSE44711 (**Figure 2B**) based on p value < 0.05 and |logFC| > 1. Two differentially expressed FRGs (HILPDA and SCD) were identified in EOPE after intersection with DEGs in GSE148241 and GSE44711 datasets (**Figure 2C**). To verify the expression of the two screened genes in the placenta of PE subtypes, we further analyzed further the expression of HILPDA and SCD in GSE74341. The GSE74341 dataset was prioritized for validation due to its ​comprehensive stratification of PE subtypes: early-onset PE (n=7, <34 weeks), late-onset PE (n=8, >36 weeks), preterm controls (n=5, <34 weeks), and term controls (n=5, >36 weeks). This design enables rigorous comparison of HILPDA expression across gestation-matched pathological and physiological contexts. The HILPDA expression in EOPE placentas was significantly increased compared to the Preterm, Term and LOPE groups (**Figure 2D**). However, the expression of SCD in the EOPE placentas showed no significant difference in either the GSE74341 dataset (vs. preterm and term placentas; **Supplementary Figure 1A**) or the GSE114691 dataset (vs. normotensive controls; **Supplementary Figure 1B**). Additionally, ROC curve analysis demonstrated that HILPDA exhibited high diagnostic accuracy for EOPE (AUC = 0.812 in GSE114691 and AUC = 0.871 in GSE10588; **Figures 2E, F**), while the AUC of SCD was 0.655 in GSE10588 (**Supplementary Figure 1C**).

### 3.2 Identification of differentially expressed genes between HILPDA-high and -low groups

**Figure 3:**
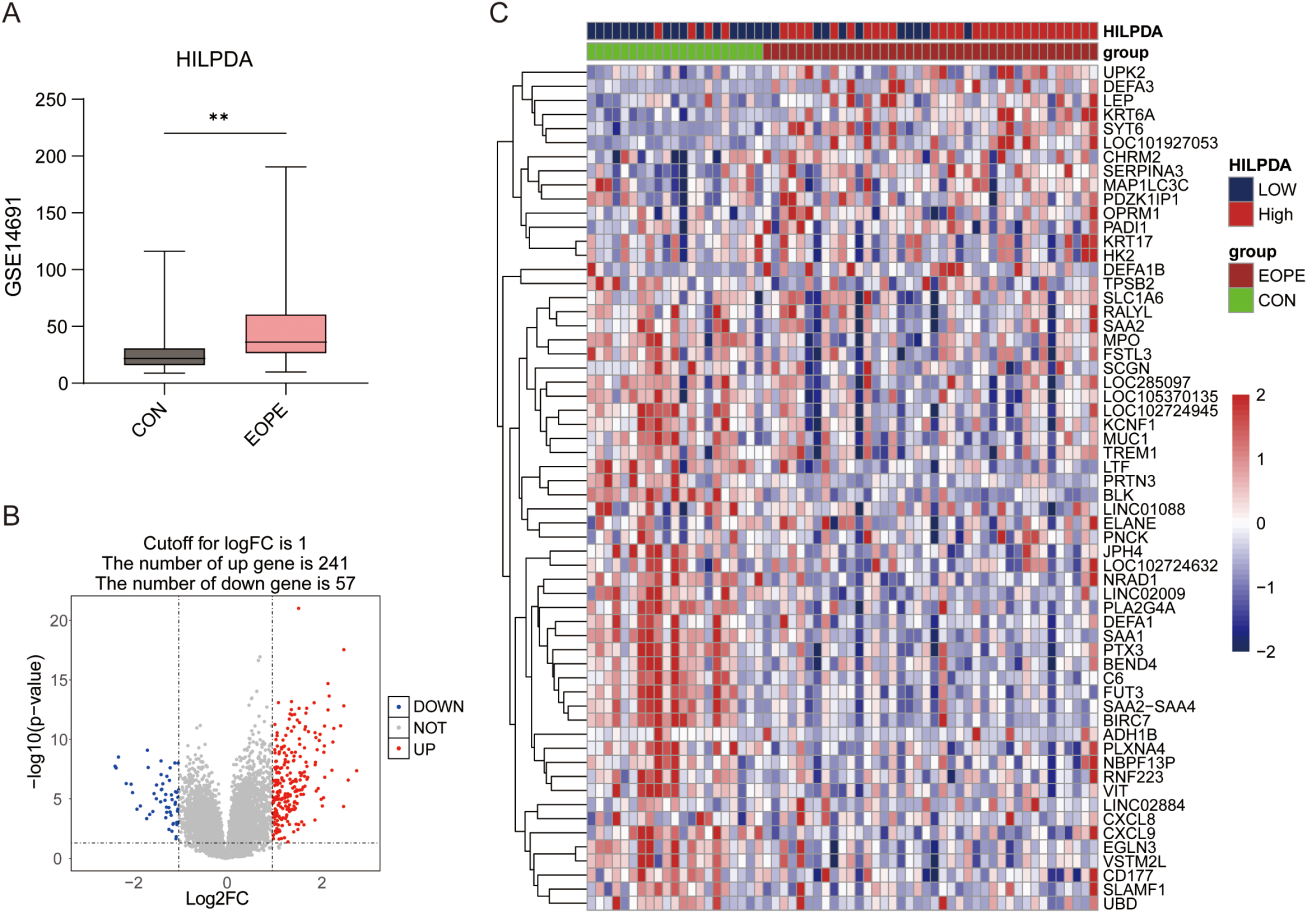
The expression of HILPDA in EOPE placentas and differential expression gene analysis between HILPDA-high and -low group. **(A)** The expression of HILPDA in EOPE placentas of GSE114691 dataset; **(B)** Volcano plot of differentially expressed genes (DEGs) between HILPDA-high and -low group in GSE114691 dataset; **(C)** A heatmap of 30 most up-regulated and 30 most down-regulated genes between HILPDA-high and low group; ^**^ *P* less than 0.01.

Given the elevated HILPDA expression in EOPE placentas and its strong diagnostic performance, additional genes correlated with HILPDA expression and associated with EOPE were identified in GSE114691 datasets. Stratification of GSE114691 samples (n=61) by median HILPDA expression yielded 33 HILPDA-high and 28 HILPDA-low placentas. As shown in **Figure 3A**, in the GSE114691 dataset, HILPDA expression was significantly upregulated in EOPE placentas, consistent with results from the GSE148241 and GSE44711 datasets. Compared to the HILPDA-low expression group, 298 DEGs were identified in the HILPDA-high samples (logFC > 1 and p value < 0.05), comprising 241 upregulated and 57 downregulated genes (**Figure 3B**). The 30 most significantly upregulated and 30 downregulated genes (ranked by log2FoldChange) were displayed in a heatmap (**Figure 3C**). These findings suggested HILPDA may take important roles in regulating placenta transcriptome.

### 3.3 Functional enrichment analysis of genes associated with HILPDA expression

**Figure 4:**
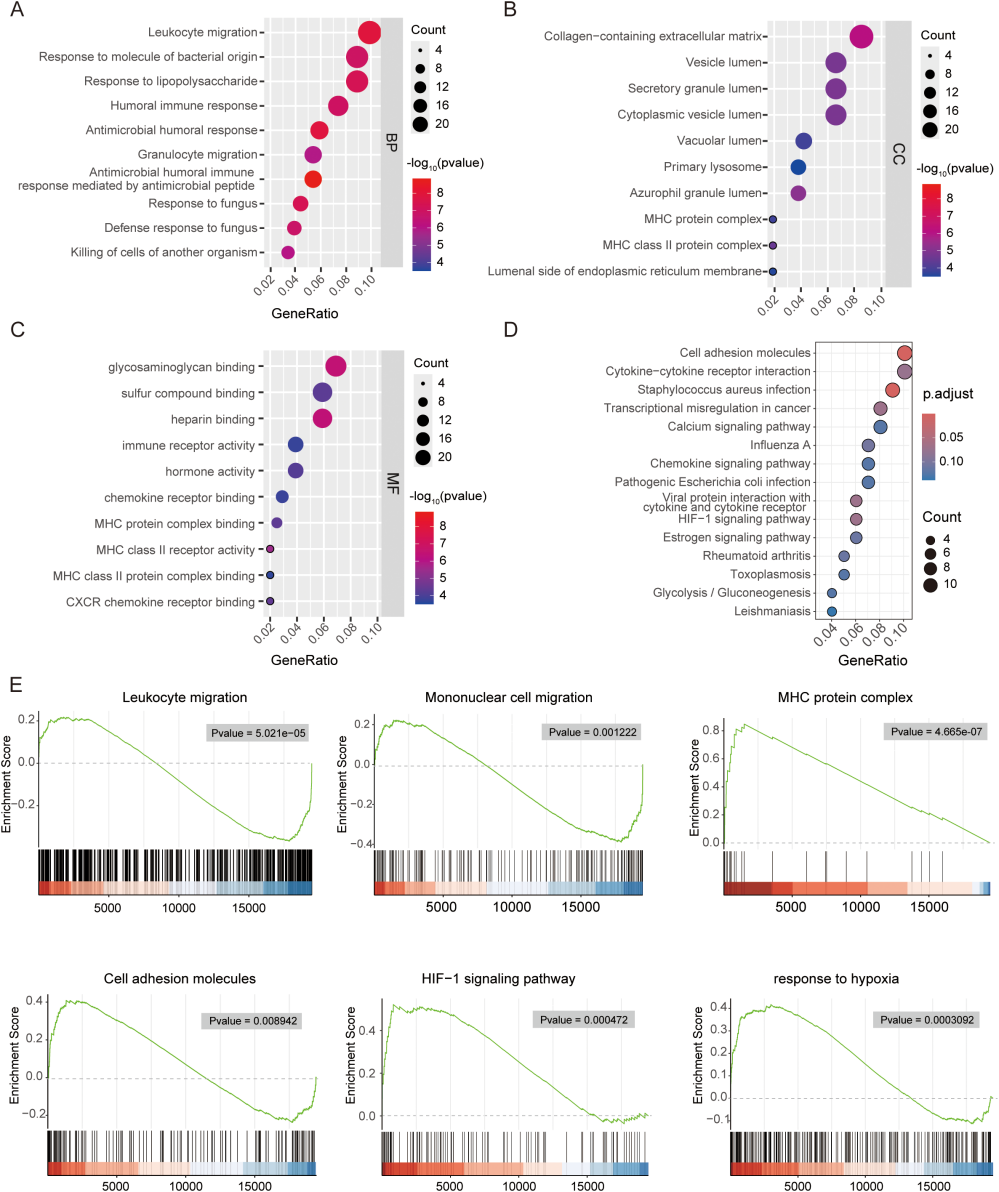
Functional enrichment analysis of differentially expressed genes between HILPDA-high and -low group. Bubble plot showing the gene ontology (GO) analysis of biological process (BP) **(A)**, cellular component (CC) **(B)** and molecular function (MF) **(C)** for upregulated genes between HILPDA-high and –low group. **(D, E)** Enrichment analyses via gene set enrichment analysis for HILPDA-related genes.

Functional enrichment analyses of all 298 DEGs between HILPDA-high and HILPDA-low groups were systematically conducted to elucidate the biological significance of HILPDA-associated genes. GO enrichment plots for BP, CC, and MF terms are shown in **Figures 4A–C**, which revealed significant enrichment of differentially expressed genes (DEGs) between HILPDA-high and -low group across all three GO domains. The biological process (BP) category revealed prominent involvement in leukocyte migration and bacterial molecular pattern response, while cellular component (CC) analysis identified enrichment in collagen-rich extracellular matrix and MHC protein complexes (**Figures 4A, B**, **Supplementary Figure 2**). Molecular function (MF) characterization further showed significant enrichment in immune receptor binding activity and humoral immune mediators (**Figure 4C**, **Supplementary Figure 2**). Furthermore, the KEGG pathway analysis revealed that DEGs between HILPDA-high and -low group were significantly involved in pathways including cell adhesion molecules and HIF−1 signaling pathway (**Figure 4D**). The results from GSEA showed that biological processes leukocyte migration and mononuclear cell migration were significantly enriched in HILPDA-low samples, while cell adhesion molecules, HIF-1 signaling pathway, and response to hypoxia pathway significantly enriched in HILPDA-high samples (**Figure 4E**). The enrichment of these pathways (immune dysregulation, matrix dysmorphia, and hypoxia adaptation) suggested HILPDA’s role in EOPE pathogenesis.

### 3.4 WGCNA-based identification of genes associated with EOPE and HILPDA expression

**Figure 5:**
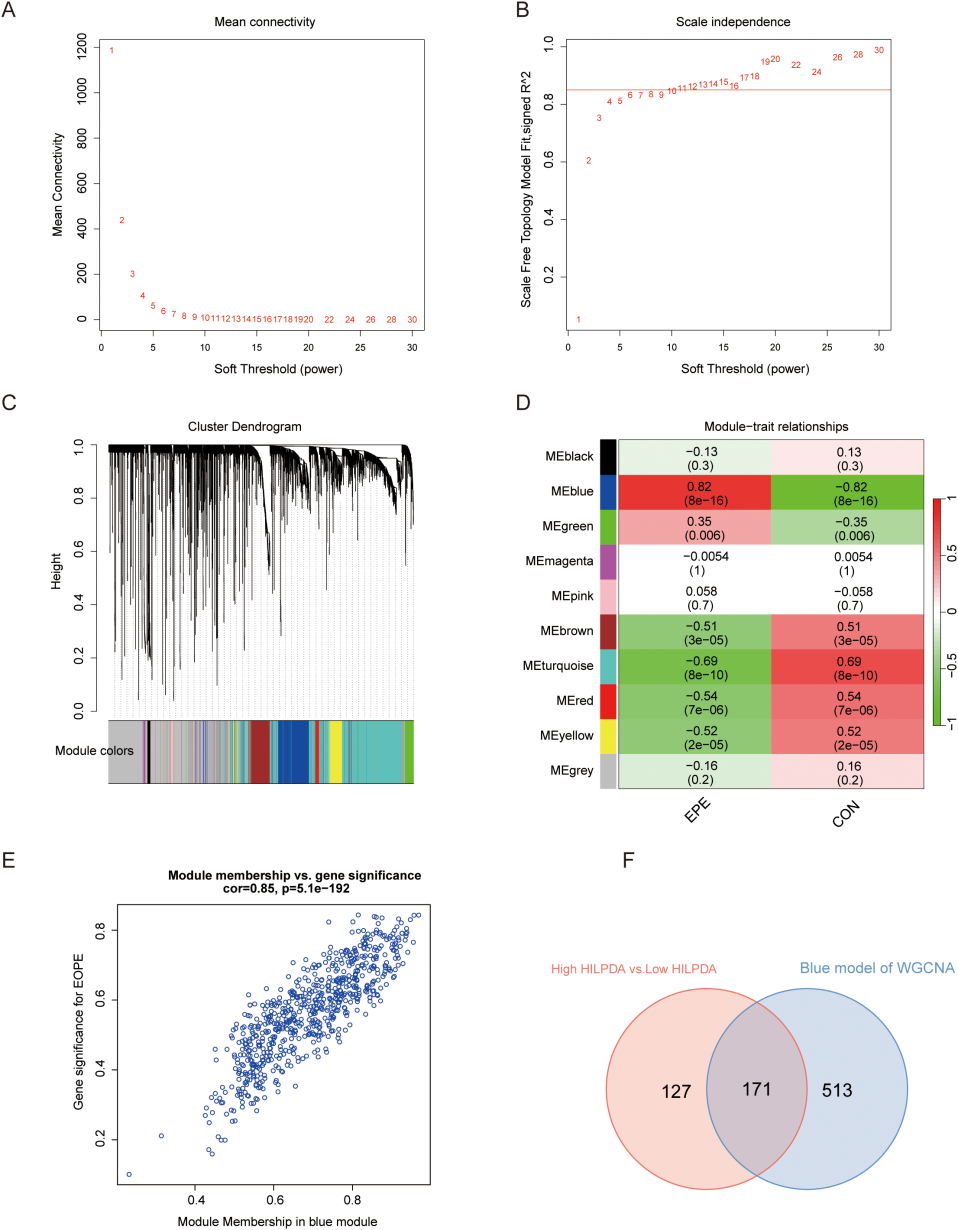
Weighted correlation network analysis (WGCNA) for screening the genes related to EOPE and HILPDA expression. **(A, B)** WGCNA with 0.85 and 11 as adjacency cutoff value and soft threshold, respectively; **(C)** The co-expression gene dendrogram and modules with different color; **(D)** The heatmap showing the correlation between gene modules and EOPE; **(E)** Correlation analysis of genes and EOPE within the blue module. **(F)** Venn diagram of the blue module and differentially expressed genes between HILPDA-high and -low group in GSE114691 dataset.

To determine the gene sets closely associated with EOPE, WGCNA was performed on the top 5,000 most variably expressed genes (selected by median absolute deviation to capture biologically relevant signals) across GSE114691 samples (n=61). As shown in **Figures 5A, B**, a soft threshold of 11(Minimum value achieving scale-free topology and lower powers compromise network biological validity) was employed to achieve an adjacency cutoff value of 0.85. After setting the minimum gene module size to 30, a total of ten modules were identified (**Figure 5C**), with the blue module (enriched in immune regulation genes) showing the strongest positive correlation (cor = 0.85, *P* = 5.1×10^-192^) with EOPE status​. The overlap between genes in the blue module and DEGs in the HILPDA-low/high groups included 171 genes, potentially identified as EOPE-correlated genes associated with HILPDA expression (**Figure 5F**).

### 3.5 Integration of multi-machine learning approaches for biomarker identification

**Figure 6:**
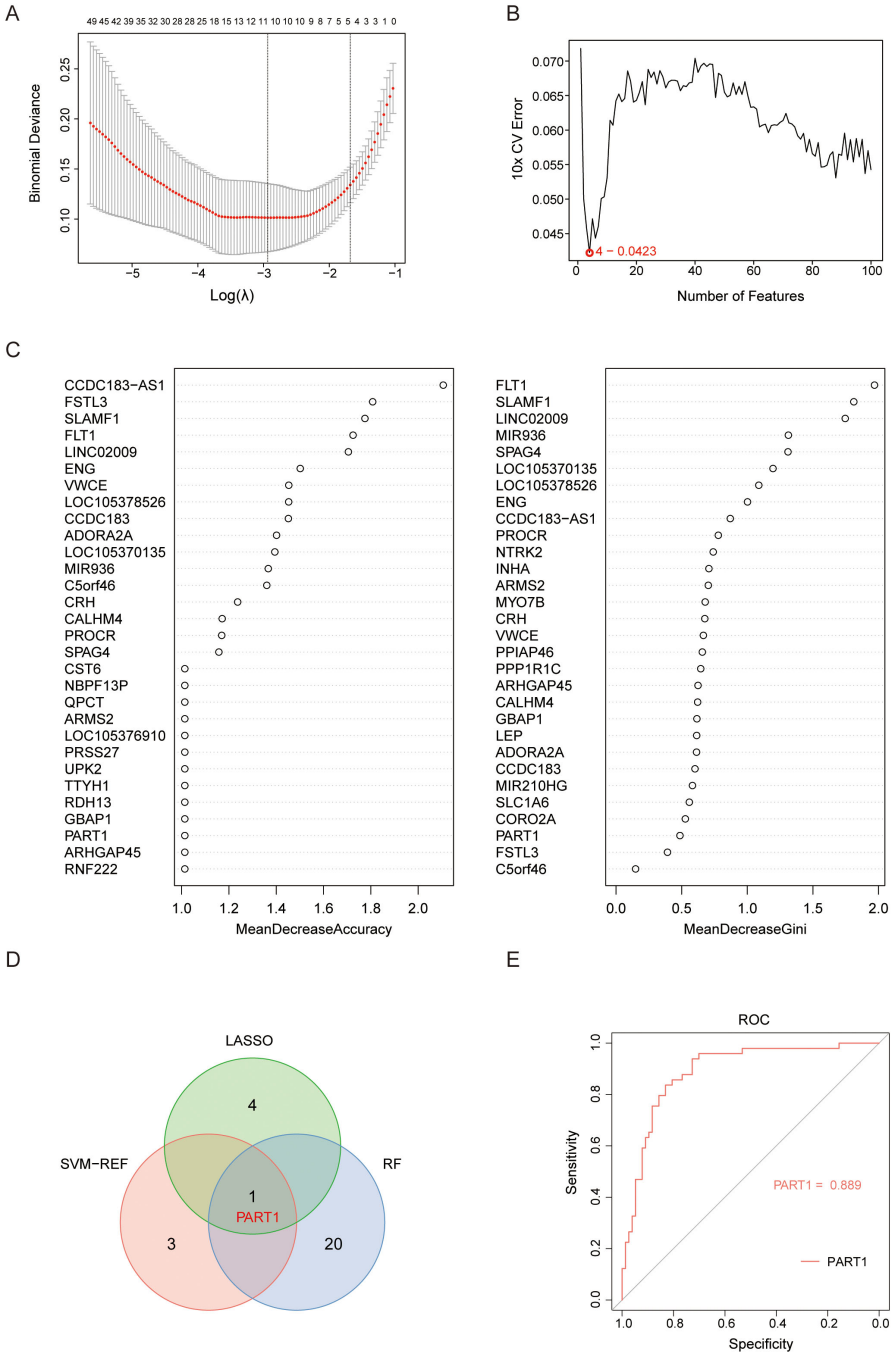
Selection of candidate diagnostic biomarkers of EOPE from HILPDA-related and EOPE-related genes with machine learning approaches. **(A)** Constructed a coefficient path plot of regularization parameter λ through 10 folds cross-validation to determine the optimal λ; **(B)** Identified 4 diagnostic biomarkers using the SVM-RFE algorithm with recursive feature elimination based on SVM weight ranking; **(C)** Ranked 30 genes by importance scores (%IncMSE or Gini index) derived from random forest; **(D)** The Venn diagram showed the intersection of candidate gene PART1 selected by LASSO, SVM-RFE, and random forest; **(E)** ROC analysis of the PART1 in GSE75010 to validate their diagnostic efficacy for EOPE.

Three machine learning models were trained on the 171 HILPDA-associated genes using GSE114691 samples with stratified 10-fold cross-validation (repeated 5 times) to maintain class balance. LASSO regression (glmnet v4.1-8) analysis (lambda.1se = 0.186) identified 5 candidate diagnostic biomarkers (PART1 NRAD1, HTRA1, LOC107985906, HTRA4) (**Figure 6A**). Subsequent application of SVM-RFE with 10-fold cross-validation revealed 4 high-confidence genes (LOC105369957, PART1, MYO7B, CLDN9) demonstrating optimal classification performance (**Figure 6B**). Finally, parallel random forest analysis (1000 decision trees, Mean Decrease Accuracy (ranked exclusively by Mean Decrease Accuracy) and Gini impurity criterion) independently prioritized 21 candidate genes through variable importance scoring (**Figure 6C**). Intersection analysis of the three method-specific candidate pools (LASSO:5, SVM-RFE:4, RF:21) identified one consensus biomarker PART1 (**Figure 6D**). PART1 is a long noncoding RNA that has been reported to has important role in regulating cell proliferation and invasion (24, 25). We validated the diagnostic performance of the identified biomarker PART1 using the independently selected external dataset GSE75010. Within this dataset, PE patients with a gestational age of less than 34 weeks were designated as the EOPE group. The ROC curve for PART1 was generated using raw expression values on a GSE75010 (49 EOPE, 77 controls) and present a good diagnostic performance for EOPE (AUC=0.889; 95% CI: 0.831-0.948) (**Figure 6E**).

### 3.6 Placental immune landscape of EOPE and correlation analysis between HILPDA and infiltrating immune cells

**Figure 7:**
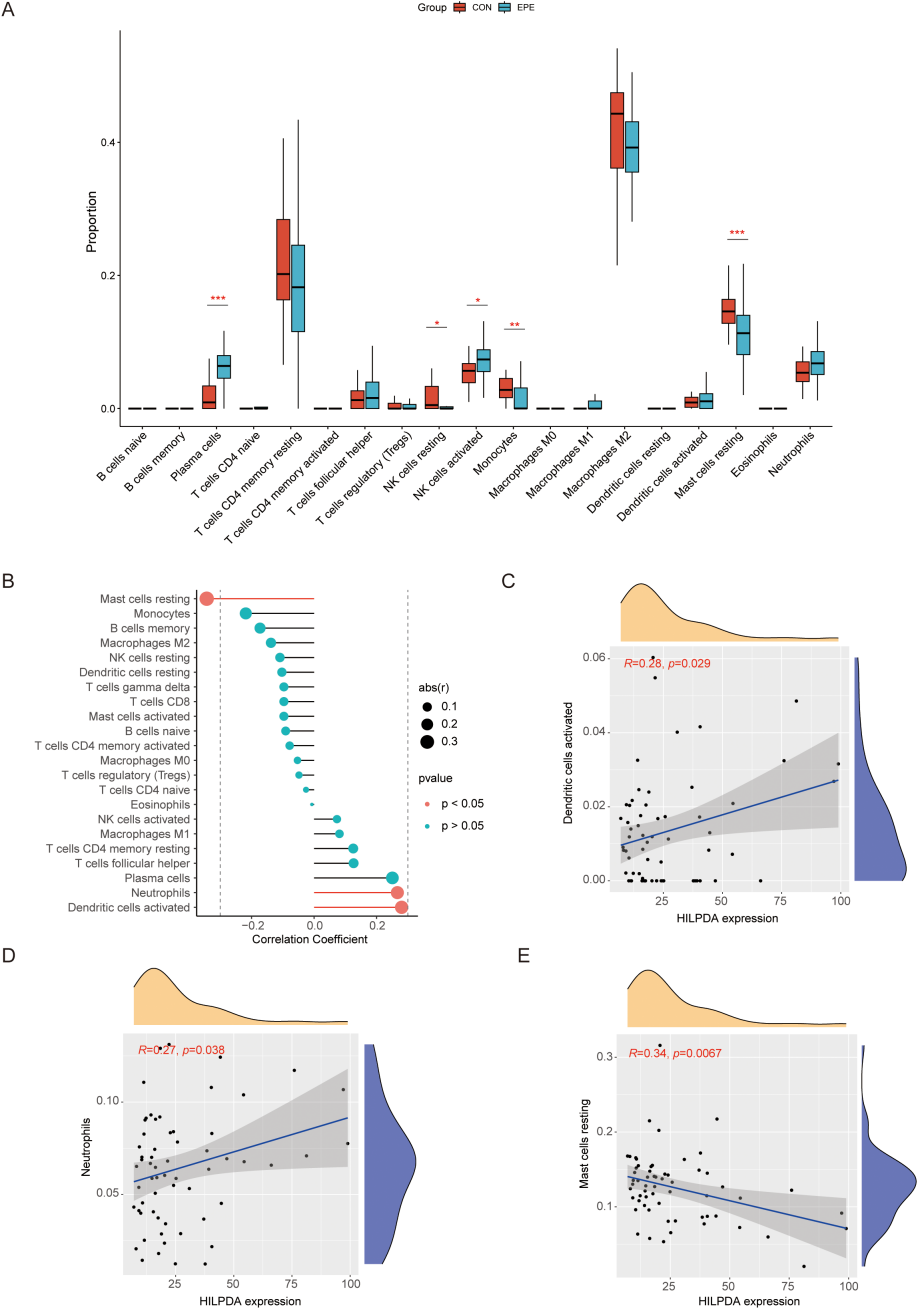
Comprehensive immune infiltration landscape in normal vs. EOPE placentas and HILPDA correlation with immune cell infiltration. **(A)** Violin diagram for immune cell distribution in normal vs. EOPE placentas; **(B)** Lollipop diagram for HILPDA expression correlation with immune cell infiltration. **(C)** Scatter plots for correlation between HILPDA expression and activated dendritic cells; **(D)** Scatter plots for correlation between HILPDA expression and neutrophils; **(E)** Scatter plots for correlation between HILPDA expression and mast cells resting. *P less than 0.05; **P less than 0.01; ***P less than 0.001.

Utilizing the CIBERSORT deconvolution algorithm, we quantitatively assessed immune cell infiltration patterns in normal and EOPE placentas (**Supplementary Figure 4**). Comparative analysis revealed distinct immunological profiles between groups (**Figure 7A**). Specifically, EOPE placentas exhibited significantly elevated proportions of plasma cells and activated NK cells compared to normal counterparts. Conversely, normal placental tissues demonstrated higher infiltration levels of resting NK cells, monocyte, and resting mast cells (**Figure 7A**). Furthermore, Spearman’s rank correlation analysis uncovered significant associations between HILPDA expression and specific immune subsets (**Figures 7B–E**, **Supplementary Figure 5**, **Supplementary Table S4**). Notably, HILPDA demonstrated a strong positive correlation with activated dendritic cells and neutrophils, while showing an inverse relationship with resting mast cells.

### 3.7 Validation of HILPDA expression in EOPE placentas and its role in regulating trophoblasts cell migration

**Figure 8:**
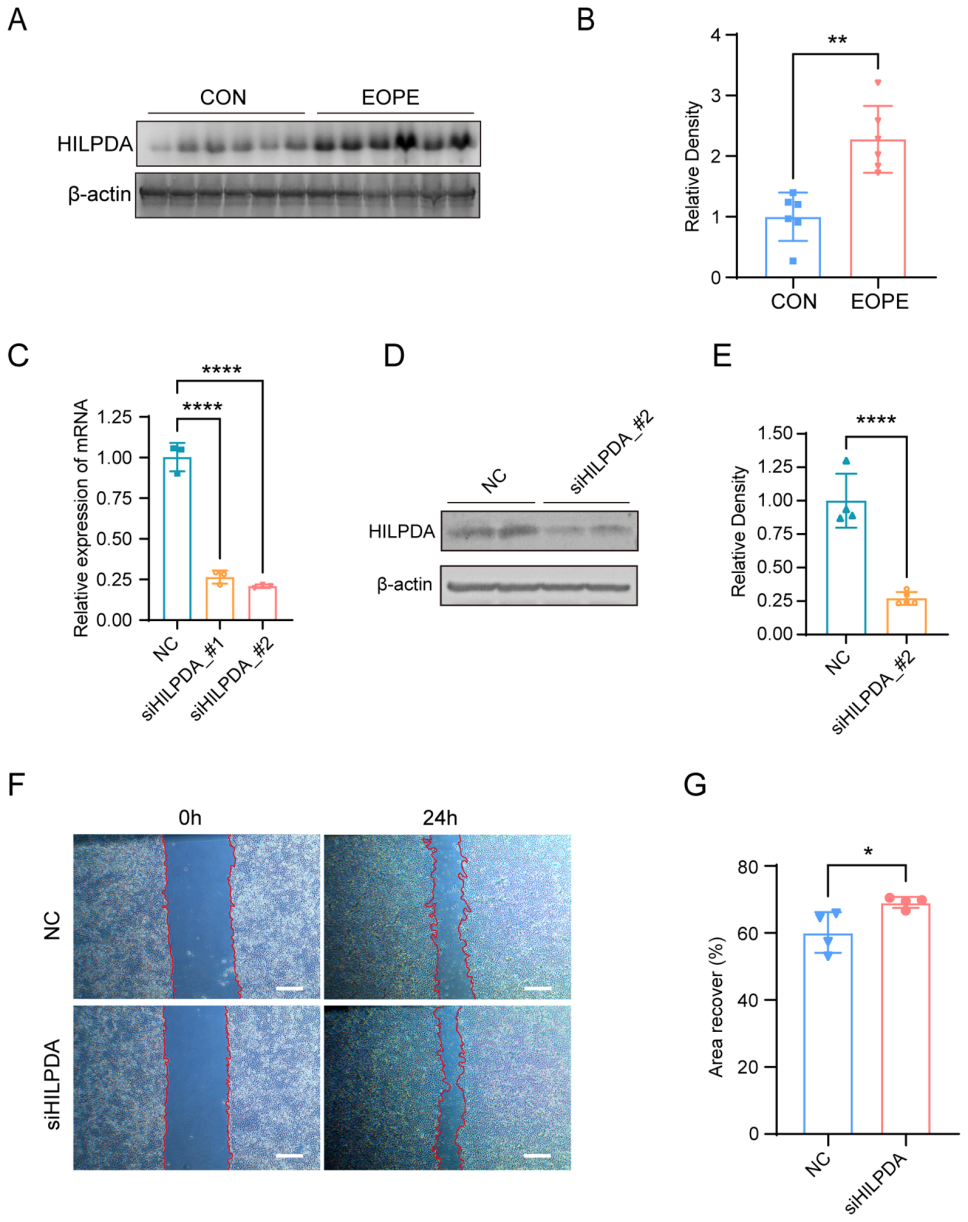
HILPDA expression in normal vs. EOPE placentas and its role in HTR-8/SVneo cell migration. Western blot analysis **(A)** and quantification **(B)** for HILPDA expression in normal (n=6) and EOPE placentas (n=6). **(C)** RT-qPCR for HILPDA mRNA levels in cells transfected with siHILPDA and negative control (NC) siRNA; Western blot analysis **(D)** and quantification **(E)** for HILPDA mRNA levels in cells from siHILPDA and NC group. Wound healing assay **(F)** and quantification **(G)** for cell migration in cells from siHILPDA (n=3) and NC group (n=3, Scale bar: 400 μm); ^*^ *P* less than 0.05; ^**^ *P* less than 0.01; ^****^ *P* less than 0.0001.

To evaluate the differential expression of HILPDA in placental pathophysiology, western blot analysis of 6 biological replicates per group was performed in placental tissues from EOPE and normotensive pregnancy. As shown in **Figures 8A, B**, western blot analyses showed that the levels of HILPDA (band intensities normalized to β-actin) were markedly elevated in EOPE placentas compared to those from normal pregnancies. Furthermore, to investigate the effect of HILPDA on trophoblast cell migration, siRNAs were used to silence HILPDA expression in HTR-8/SVneo cells and the result showed a good silencing efficiency of HILPDA at both the mRNA and protein levels (**Figures 8C–E**). Wound healing assays quantified at 24 hours post-scratch demonstrated significantly enhanced migration in HILPDA-knockdown cells versus scrambled siRNA controls (**Figures 8F, G**). These findings suggested that HILPDA critically suppresses trophoblast migration capacity, and its pathological overexpression in EOPE placentas may directly contribute to impaired spiral artery remodeling, a hallmark defect in EOPE pathogenesis.

## 4 Discussion

PE is a serious complication that affects both the mother and the unborn baby, potentially leading to life-threatening issues if not managed properly. EOPE and LOPE are subtypes of PE distinguished by their onset during pregnancy. Although both subtypes cause similar vascular problems, their pathogenesis and severity differ significantly. EOPE typically results from poor placental development early in pregnancy, causing inadequate blood supply and systemic issues such as inflammation and high blood pressure. In contrast, LOPE usually stems from maternal factors in later pregnancy stages, such as an inability to meet increased metabolic demands, leading to milder symptoms due to better placental development (2, 3). Our previous study indicated that EOPE and LOPE have distinct underlying molecular mechanisms, with ferroptosis playing a more prominent role in the pathogenesis of EOPE (26).

In this study, we systematically analyzed the expression of ferroptosis-related genes in the EOPE placentas from various GEO datasets and identified HILPDA as a crucial ferroptosis-related gene in EOPE through multi-dataset analysis. Specifically, differential expression analysis across placental samples (GSE148241 and GSE44711) revealed 2 ferroptosis-related DEGs (HILPDA and SCD), with HILPDA showing consistent upregulation in EOPE across validation datasets (GSE74341, GSE114691, and GSE10588). HILPDA demonstrated superior diagnostic performance (AUC=0.871). Functional analysis of HILPDA-associated genes identified enrichment in immune regulation (leukocyte migration, MHC complexes) and cellular processes (collagen matrix organization, HIF-1 signaling pathway). WGCNA identified 171 HILPDA-associated DEGs exhibiting strong correlation with EOPE pathogenesis. Machine learning integration (LASSO, SVM-RFE, Random Forest) identified one high-performance diagnostic biomarker PART1 (AUC=0.993) from the 171 HILPDA-associated DEGs. Immune profiling showed EOPE placentas had increased plasma cells/activated NK cells and revealed HILPDA’s correlation with dendritic cell activation. Experimental validation confirmed HILPDA overexpression in EOPE placentas and demonstrated its regulating role in trophoblast migration through siRNA knockdown in HTR-8/SVneo cells. The findings position HILPDA as a key regulator of placental immune dysregulation and trophoblast migration in EOPE pathogenesis.

In a healthy pregnancy, the maternal immune system undergoes adaptations to tolerate the semi-allogeneic fetus (27). Leukocytes, including macrophages, T cells, and dendritic cells, are crucial for these immune adaptations. In PE, there are evidences of increased leukocyte activation and altered migration patterns. For example, pro-inflammatory cytokines were found to be present in higher concentrations in women with PE (28); Activation of neutrophils and monocytes occurs in preeclamptic pregnancies, but not in normal pregnancies (29).

HILPDA is a protein associated with lipid droplets and regulated by hypoxia. Recently, increasing studies suggested that it plays several roles in immune regulation beyond its established function in lipid metabolism. The altered lipid metabolism due to HILPDA deletion may change the distribution and activity of T cells and B cells in the liver, altering immune response patterns (30). The other study demonstrates that HILPDA upregulate IL-10 to activate the STAT3 signaling pathway in NK cells, which promotes hepatocellular carcinoma immune evasion and progression (31). In breast cancer metastasis models, HILPDA expression in pulmonary mesenchymal cells promote lipid accumulation and metabolically reprogram lung-resident NK cells leading to NK cell dysfunction (32). This evidence positions HILPDA as a molecular mediator bridging immune-stromal crosstalk, actively reprogramming the tumor microenvironment through lipid metabolic remodeling. To further investigate the role of HILPDA in EOPE, we performed a functional enrichment analysis on the genes correlated with HILPDA expression and associated with EOPE. The results showed that EOPE- and HILPDA-related genes are significantly involved in the regulation of immune including leukocyte migration, mononuclear cell migration and MHC complexes. Moreover, our immune functional enrichment analysis further suggests that HILPDA may mediate abnormal immune cell infiltration in the placenta. The observed increase in activated NK cells and plasma cells in EOPE placentas indicates that the upregulation of HILPDA may contribute to the formation of a localized inflammatory environment, thereby disrupting maternal-fetal immune tolerance and normal placental function. While our immune correlation analyses and other studies in cancer suggest HILPDA may modulate NK cell dynamics, the HILPDA’s role in placental immunobiology requires direct experimental validation in pregnancy-specific contexts since the different immunosuppressive microenvironment may exist between placenta and tumors.

At the same time, the integration of multiple datasets and machine learning approaches allowed us to identify one HILPDA-associated DEG PART1 as a potential high-performance diagnostic biomarker. This not only further underscores the importance of HILPDA in the pathogenesis of EOPE but also provides new molecular evidence for early diagnosis. The high diagnostic accuracy of the biomarker (with AUCs of 0.889) indicates that they hold promise for clinical use in assessing EOPE risk, thereby facilitating more precise individualized management and early intervention.

It is well known that EOPE arises from ​defective placental development​ initiated by impaired extravillous trophoblast invasion into maternal decidua, resulting in ​incomplete spiral artery remodeling. This leads to placental hypoperfusion and ischemia-reperfusion injury triggering a cascade of pathological events such as hypoxic stress, oxidative damage​, immune dysregulation and ​endothelial dysfunction​ via VEGF signaling disruption (33). Trophoblast migration and invasion are essential for complete spiral artery remodeling during placental development (34, 35). Cell-cell adhesion plays a crucial role in cell migration, influencing various biological processes such as embryonic development, wound healing, and cancer metastasis (36, 37). It ensures cells maintain proper coordination, directional movement, and effective signaling, all of which contribute to force generation and cell survival (38). In this study, our functional enrichment analysis revealed that genes associated with HILPDA are significantly enriched in pathways governing cell adhesion and extracellular matrix organization suggesting that HILPDA may play a pivotal role in modulating cell-cell adhesion dynamics. Moreover, we demonstrated HILPDA protein levels significantly increased in EOPE placentas and our cell scratch assays demonstrated that altering HILPDA expression significantly affects the migration capacity of trophoblast cells. Cells with reduced HILPDA levels exhibited increased migration ability in trophoblast cells, indicating that HILPDA may influence the reorganization of adhesion complexes or cytoskeletal structures necessary for efficient cell movement, processes critical for proper placentation and pregnancy outcomes. In the course of placentation, inadequate endovascular invasion by trophoblasts, a process dependent on migration, leaves spiral arteries constricted and hypoxic, prone to atherosis (35). HILPDA overexpression in EOPE placentas likely contributes to this pathology by hindering trophoblast motility, thereby perpetuating the ischemic placental microenvironment that characterizes early-onset disease.

Integrated evidence in this study suggests that HILPDA may coordinate hypoxia response, immune dysregulation, and impaired trophoblast function in EOPE pathogenesis. Mechanistically, placental hypoxia potentially activates HIF signaling (particularly HIF-2α), inducing HILPDA overexpression (11). Upregulated HILPDA could subsequently operate through dual pathways: (1) It remodels lipid metabolism to promote ferroptosis susceptibility (11), amplifying placental damage under iron-rich/hypoxic conditions; (2) It orchestrates immune dysregulation by modulating leukocyte migration pathways (GO analysis) and correlating with specific immune infiltrates (activated dendritic cells, neutrophils, and resting mast cells; **Figure 7**). This may create a pro-inflammatory milieu that further disrupts maternal-fetal tolerance. Crucially, HILPDA simultaneously suppresses trophoblast migration (**Figures 8F, G**), which may contribute to the abnormal spiral artery remodeling. The resulting failure in placental perfusion perpetuates hypoxia, forming a self-amplifying pathological cycle where HILPDA may integrate hypoxic stress, immune dysfunction, and cellular defects to drive EOPE progression.

Although this study provided new insights into the complex pathological mechanisms of EOPE, there were several limitations in this study. First, the promising diagnostic biomarker (PART1) identified through machine learning algorithms was validated only in silico using existing GEO datasets. Their diagnostic utility and clinical applicability require rigorous prospective validation in larger, independent patient cohorts with standardized sample collection and processing. Furthermore, while WGCNA provided high-resolution network insights, it was employed as an exploratory, unsupervised method to identify potential gene modules associated with EOPE and its results require biological validation due to sensitivity to parameter choices. In addition, the experimental validation using human placental tissues (Western blot) and functional assays (siRNA migration) involved relatively small sample sizes. While powered for initial validation, larger cohorts would strengthen the statistical robustness and generalizability of these key findings. Finally, although HILPDA knockdown demonstrated an effect on trophoblast migration *in vitro*, the precise molecular mechanisms linking HILPDA overexpression to cell migration, specific immune cell alterations, and impaired spiral artery remodeling *in vivo* remain incompletely defined. Future research should focus on elucidating the specific interactions between HILPDA and immune cells and its mechanism involved in regulating trophoblast migration, with the aim of providing theoretical and practical guidance for the development of new therapeutic strategies.

## Data Availability

The original contributions presented in the study are included in the article/**Supplementary Material**. Further inquiries can be directed to the corresponding authors.
