# Supplementary material for: Identification and validation of expression and functions of ferroptosis-related gene HILPDA in early-onset preeclampsia placentas
Source: Front Immunol. 2025 Aug 11;16:1627057. doi: 10.3389/fimmu.2025.1627057 (PMC12375643; doi:10.3389/fimmu.2025.1627057)
Supplement: Supplementary file 1 [file SupplementaryFile1.docx]

***Supplementary Information***

**1 Supplementary Tables**

Table S1: The information of GEO datasets used in this study.

| **Datasets** | **Platform** | **Number of EOPE** | **Number of controls** | **Tissue type** | **Preprocessing steps** |
| --- | --- | --- | --- | --- | --- |
| GSE148241 | Illumina HiSeq 2500  (Homo sapiens) | 9 | 32 | Placenta | Raw counts of two umbilical cord blood were used to remove the influence of blood contamination on gene expressions in placenta. |
| GSE44711 | Illumina HumanHT-12 V4.0 expression beadchip | 8 | 8 | Chorionic Villi | log2 transformation and skip missing values (removes all genes containing any missing values) |
| GSE114691 | Illumina HiSeq 2000 (Homo sapiens) | 21 | 40 | Placenta | log2 transformation  Low-count filtering (≥10 counts in ≥21 samples) |
| GSE75010 | [HuGene-1_0-st] Affymetrix Human Gene 1.0 ST Array | 49 | 77 | Placenta | RMA background correction  Quantile normalization |
| GSE10588 | ABI Human Genome Survey Microarray Version 2 | 17 | 26 | Placenta | RMA background correction  Quantile normalization |

Table S2: Clinical information of patients

| **Category** | **Normal** | **Preeclampsia** | ***P*-value** |
| --- | --- | --- | --- |
| Age (years) | 31.83±3.125 | 32.00±4.099 | 0.9348 |
| Weight (kg) | 78.33±9.993 | 79.83±9.496 | 0.7952 |
| Systolic blood pressure (mmHg) | 119.5±8.871 | 172.8±5.672 | <0.0001 |
| Diastolic blood pressure (mmHg) | 77.83±9.020 | 100.7±7.339 | 0.0007 |
| Proteinuria (%) | 0 | 100 | <0.0001 |
| Gestational age at delivery (weeks) | 38.83±0.408 | 32.83±3.312 | 0.0013 |
| Neonatal birth weight (g) | 3676±393.7 | 1898±881.1 | 0.0011 |

Values are presented as n (%) or mean±SD.

SD, standard deviation.

Table S3. siRNA sequences used for RNA interference

| **siRNA** | **Manufacturer** | **Cat.no.** | **Sequence** |
| --- | --- | --- | --- |
| siHILPDA_#1 | RIBOBIO | stB0010975A-1-5 | GTGTGGTACTGACCCTACT |
| siHILPDA_#2 | RIBOBIO | stB0010975B-1-5 | GAGTGATGGAGTCCCTAGA |
| negative control siRNA | RIBOBIO | siN0000001-1-5 | Proprietary sequence |

Table S4: Correlation between HILPDA and infiltrating immune cells

| **Cell type** | **Correlation coefficients** | **p-values** |
| --- | --- | --- |
| B cells naive | -0.091 | 0.49 |
| B cells memory | -0.17 | 0.18 |
| Plasma cells | 0.25 | 0.05 |
| T cells CD4 naive | -0.03 | 0.84 |
| T cells CD4 memory resting | 0.13 | 0.34 |
| T cells CD4 memory activated | -0.078 | 0.55 |
| T cells follicular helper | 0.13 | 0.33 |
| T cells regulatory (Tregs) | -0.05 | 0.71 |
| NK cells resting | -0.11 | 0.40 |
| NK cells activated | 0.07 | 0.57 |
| Monocytes | -0.22 | 0.09 |
| Macrophages M0 | -0.05 | 0.68 |
| Macrophages M1 | 0.08 | 0.53 |
| Macrophages M2 | -0.14 | 0.29 |
| Dendritic cells resting | -0.10 | 0.43 |
| Dendritic cells activated | 0.28 | 0.03 |
| Mast cells resting | -0.34 | <0.01 |
| Eosinophils | -0.01 | 0.96 |
| Neutrophils | 0.27 | 0.04 |

**2 Supplementary Figures**


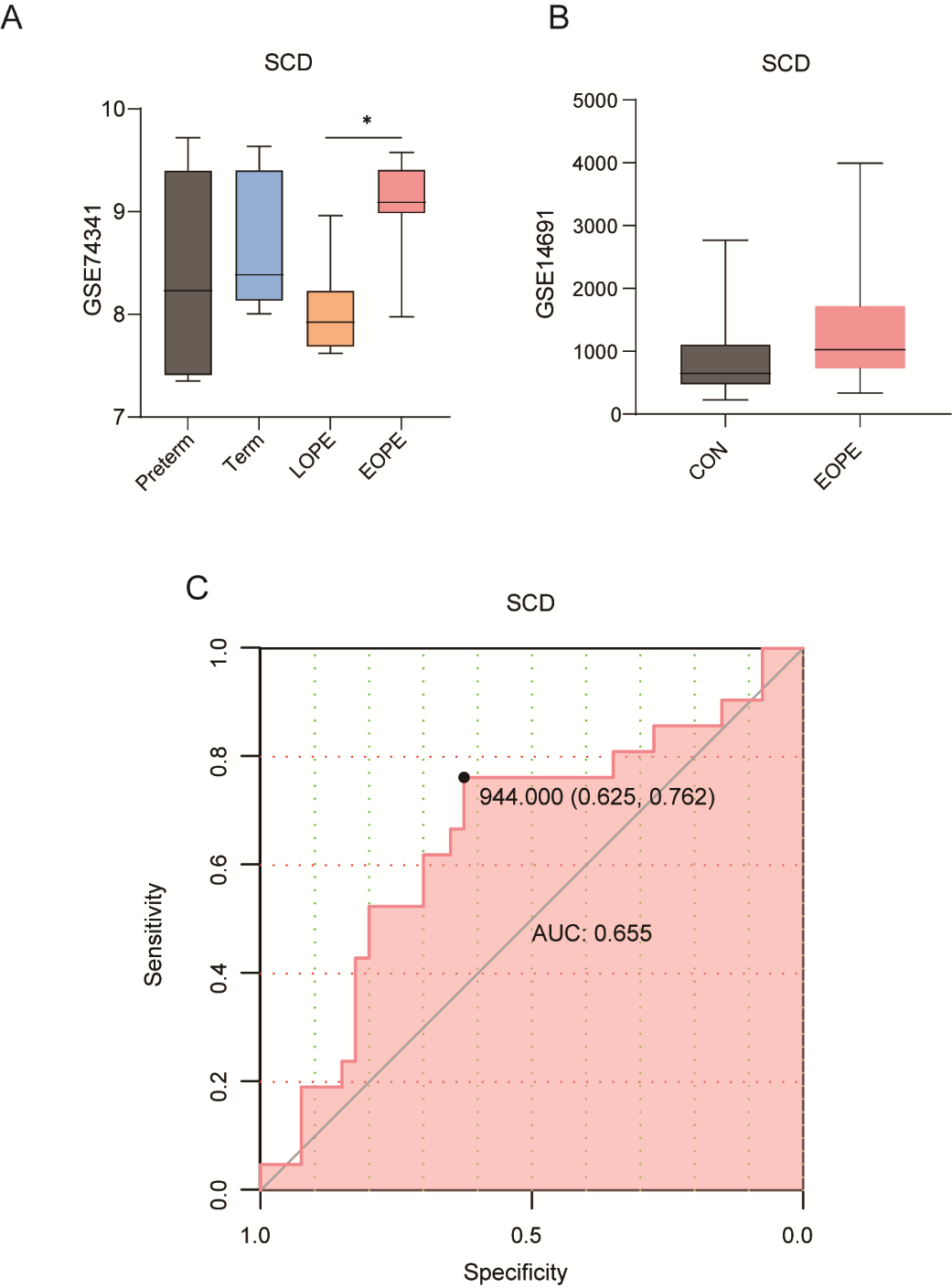


**Supplementary Fig.1 Differentially expressed ferroptosis-related genes (FRGs) and the prognostic value of SCD in EOPE.** The expression of HILPDA and SCD in placentas from preterm, term, LOPE and EOPE patients in GSE74341 **(A)** and GSE14691 **(B)**. **(C)** The ROC curve and expression of SCD in EOPE placentas of GSE14691 dataset.


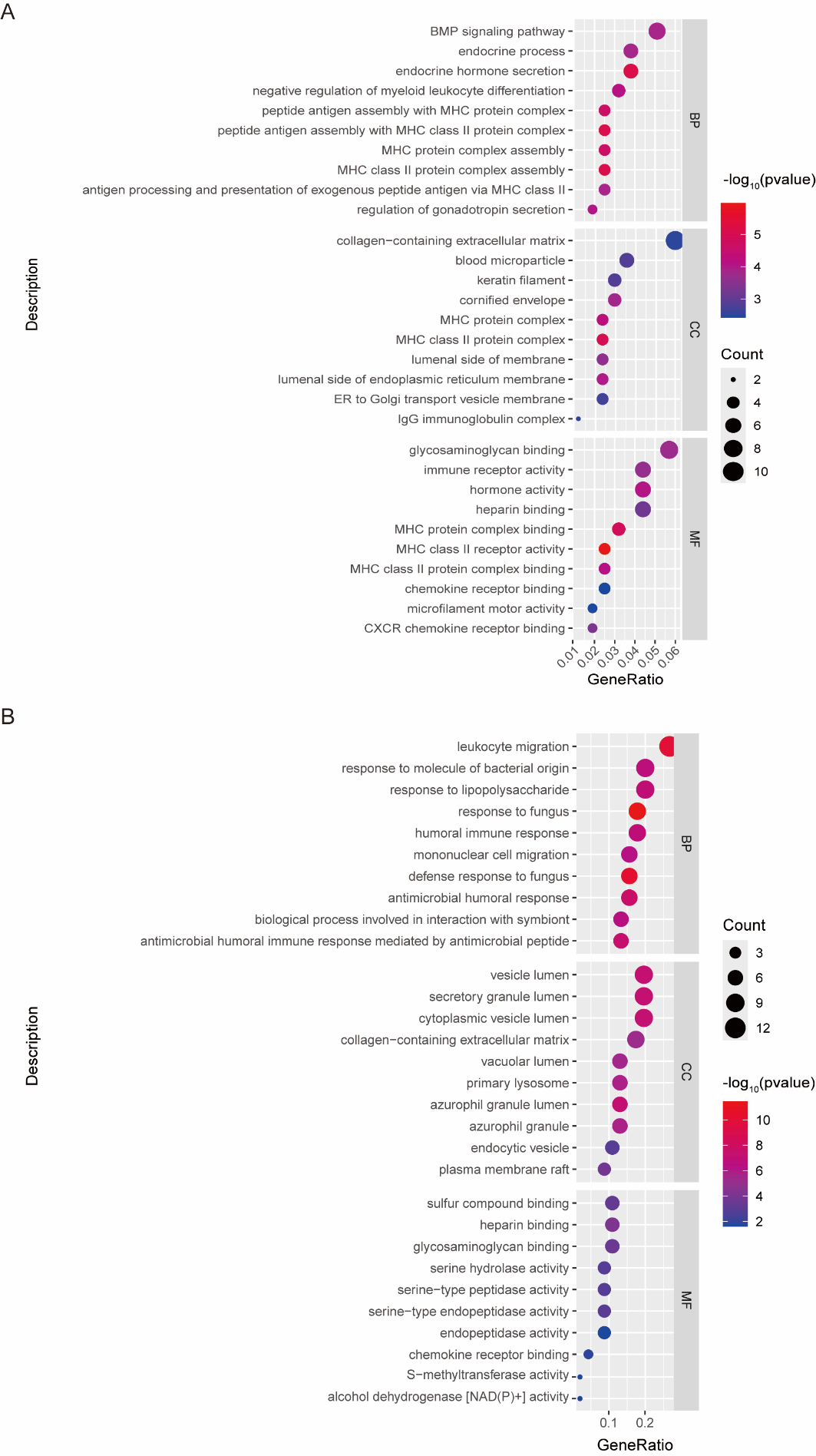


**Supplementary Fig.2 Functional enrichment analysis of genes associated and HILPDA expression. (A)** Bubble plot showing the gene ontology (GO) analysis of upregulated genes between HILPDA-high and –low group; **(B)** Bubble plot showing the GO analysis of downregulated genes between HILPDA-high and –low group;


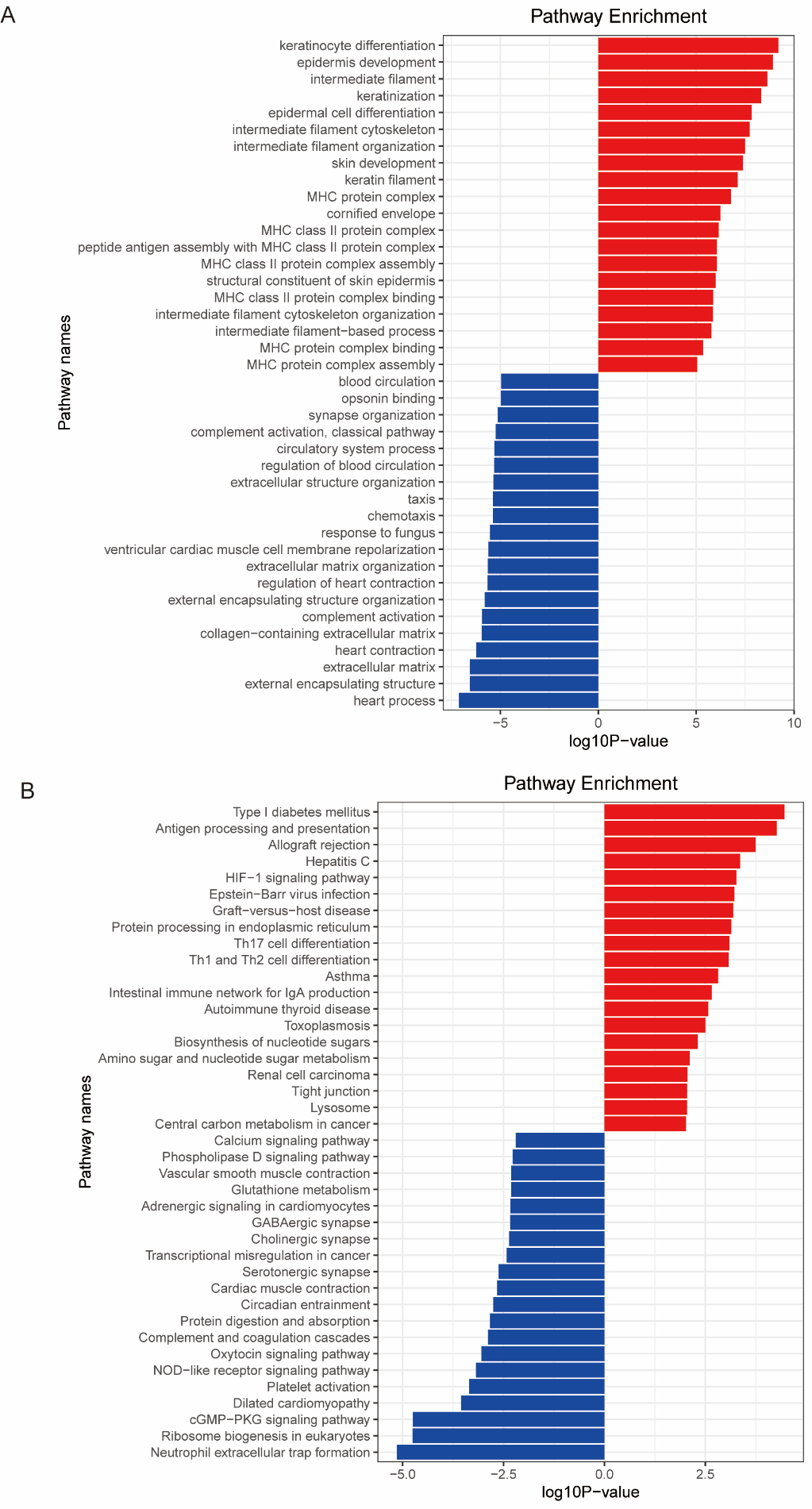


**Supplementary Fig.3 Gene Set Enrichment Analysis (GSEA ) of genes associated and HILPDA expression.** GSEA analysis of gene ontology **(A)** and Kyoto Encyclopedia of Genes and Genomes pathways **(B)** among HILPDA-associated genes.


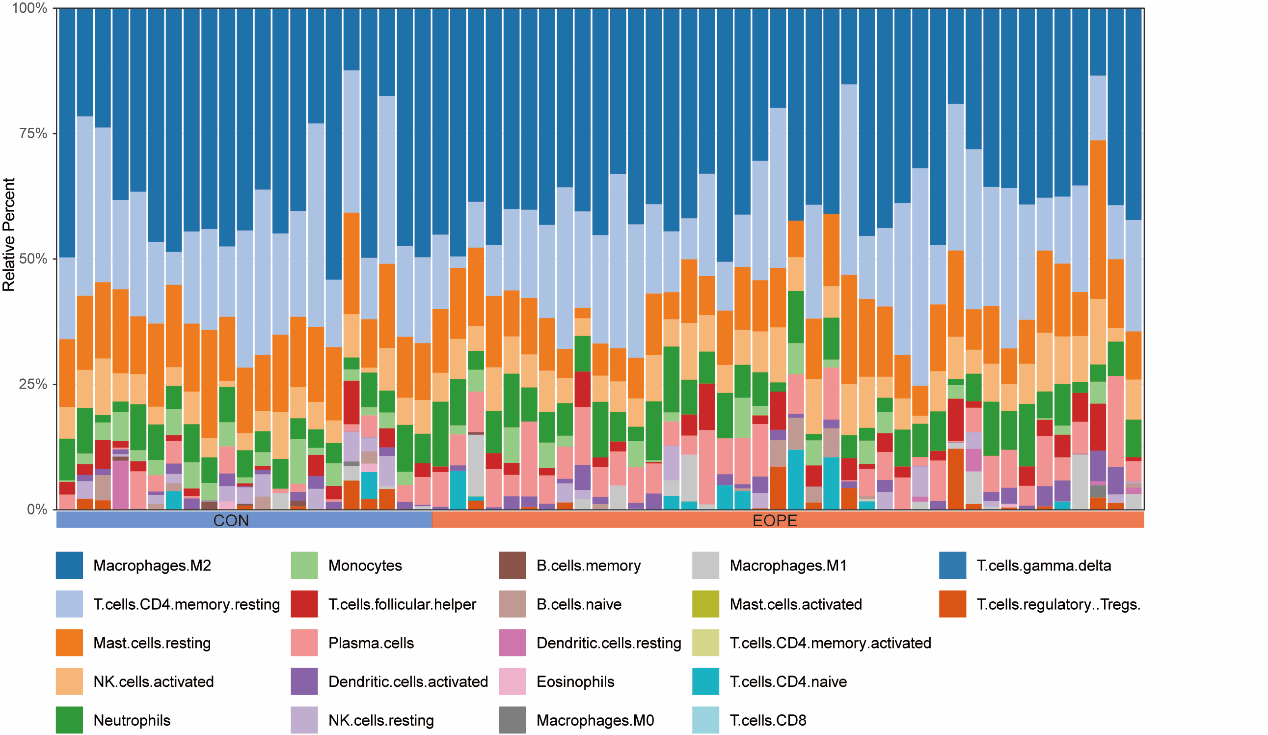


**Supplementary Fig.4 The proportion of** **infiltrating immune cells in the normal and EOPE placentas**

**
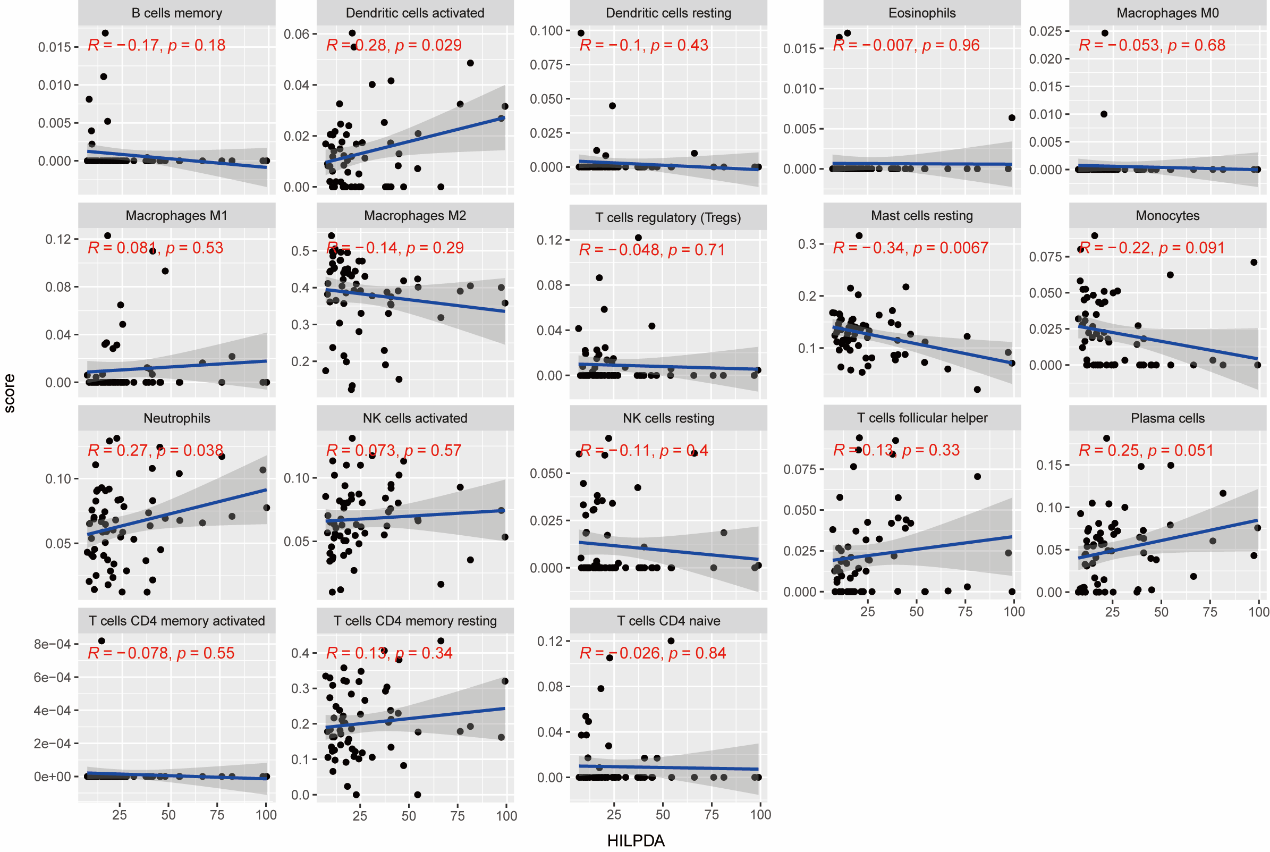
**

**Supplementary Fig.5 Scatter plots for correlation between HILPDA and immune cells in the normal and EOPE placentas**
